# Gender Bias in the Evaluation of Teaching Materials

**DOI:** 10.3389/fpsyg.2020.01074

**Published:** 2020-05-26

**Authors:** Asri Özgümüs, Holger A. Rau, Stefan T. Trautmann, Christian König-Kersting

**Affiliations:** ^1^Department of Economics, University of Göttingen, Göttingen, Germany; ^2^Department of Economics, Heidelberg University, Heidelberg, Germany; ^3^Department of Economics, Tilburg University, Tilburg, Netherlands; ^4^Department of Banking and Finance, University of Innsbruck, Innsbruck, Austria

**Keywords:** gender equality, discrimination, teaching evaluations, higher education, experiment

## Abstract

Gender differences in university teaching evaluations are well established, showing less favorable assessments of female instructors. It has also been shown that these differences cannot be linked to differences in students’ course performance, which would justify differences in evaluations. The less favorable assessments are thus either due to differences in aspects that do not affect student performance, but do affect their class experience (e.g., likability of voice tone), or due to evaluation biases unrelated to any actual differences in class experience. We find support for the latter mechanism when any differences between instructors are excluded by having respondents judge identical teaching materials prepared by either a male or a female instructor. In two studies, we find that female instructors receive worse ratings than male instructors from male respondents. In one study, we also find that female instructors receive higher ratings from female raters. Gender bias vanishes for non-academic subjects in our data.

## Introduction

Glass Ceiling effects are a severe problem in labor markets (Bagues and Esteve-Volart, 2010; Christofides et al., 2013), with the underrepresentation of women in executive positions sometimes linked to the widely discussed gender pay gap (Blau and Kahn, 1994; Blau and Kahn, 2017; Petrongolo, 2019). An important question therefore concerns the underlying reasons for these glass ceiling effects. Supply-side issues may be responsible for the phenomenon. An example may be maternity leave as an important contributor to career breaks and glass ceiling effects (Cabeza et al., 2011). If, however, gender differences in preferences are relevant (Croson and Gneezy, 2009), women may be less competitive and may “shy away” from executive positions (Niederle and Vesterlund, 2007). Another source may be related to the demand side, with employers deciding about hiring or promotion possibly implicitly discriminating against women. Evaluation processes may play an important role in this case. For instance, Goldin and Rouse (2000) show for audition procedures of symphony orchestras that the likelihood of women advancing increases when blind auditions are applied. Biernat et al. (2012) find systematically higher performance ratings for male than for female attorneys by male supervisors in a Wall Street law firm. Similar labor market patterns are also observed in academia, where women are underrepresented at tenured faculty positions in many fields (Mengel et al., 2019). In the field of academia, evaluation processes play a key role for career advancement, and subtle biases in such processes may be part of an explanation of worse outcomes for female scholars (conditional on a candidate’s quality). Although Ceci and Williams (2011) review argues that institutional aspects may be more important for gender differences in career outcomes in academia, several recent studies have shown that subtle biases do exist. Examples include the assessment of scientific papers (Budden et al., 2008; Krawczyk and Smyk, 2016), the assessment of grant proposals for research funding (Bornmann et al., 2007), and potential discrimination in teaching evaluations (Boring, 2017; Mengel et al., 2019), which is the focus of the current study.

Potential gender bias in teaching evaluations may have implications for academic careers and faculty composition. However, while it is easy to compare evaluations of male and female instructors, it is difficult to identify a bias in such evaluations. If male instructors receive higher evaluations, this may indeed be due to better teaching performance. Alternatively, it may be due to a subjectively better class experience by participants (voice tone, body language, personality), even if unrelated to teaching effectiveness. A recent paper by Mengel et al. (2019) used a large dataset on randomly assigned tutorial groups to demonstrate that female instructors are rated lower than male instructors, and that these evaluations do not correlate with teaching effectiveness measures. However, the study cannot distinguish whether differences in evaluations emerge because of differences in the subjective experience of the evaluators, or whether there is a gender bias unrelated to any factual class experience. Although both phenomena can be construed as a bias, they arise from different sources, and need to be addressed in different ways. For example, it may be less clear how to approach gender differences emerging from differences in actual class experience.

The current study aims at separating these two explanations, which requires holding any subjective experiences exactly identical across male and female stimuli. Mengel et al. (2019) find that identical teaching material is evaluated differently depending on the instructor’s gender. However, they argue that the difference in judgment of the teaching performance as a whole might have influenced the assessment of the teaching materials. MacNell et al. (2015) employ an online course format in which they assign a male and a female instructor once to their own identities and once to the identity of another person. Thus, they compare evaluations of the same person acting once as a male and once as a female. These authors find no overall differences in evaluations of the male and the female instructor by true gender, but they find that the female online identities received lower evaluations. The result therefore provides evidence for a gender bias because the teaching experience is held constant as online gender variation is within-person. Although the study makes an important contribution, a potential problem with MacNell et al.’s (2015) study is that construct validity is threatened by an inappropriate sampling of stimuli: first, only one male and one female identity are used as stimuli. Second, the identity of the stimuli is not experimentally manipulated because the true course instructors had to be used as stimuli. As demonstrated by Wells and Windschitl (1999), having a single stimulus representing a category (e.g., males) can lead to a confounding of the characteristics of the selected stimulus with the category. We may then misinterpret the effect of the unique stimulus characteristics as an effect of the characteristics of the category. Hence, it is possible that other aspects of the female stimulus’ online name, rather than gender, influenced the evaluations relative to the male stimulus’ name (e.g., by names signaling education, or race, or age). Moreover, MacNell et al.’s (2015) sample is rather small with 43 self-selected participants who potentially know the instructors from other interactions, amplifying this problem.

Other studies have employed methodology that can identify gender bias independently of subjective experiences. For example, Arbuckle and Williams (2003) have students evaluate the sound recording of a lecture held in a gender-neutral voice, telling the participants that the speaker is either male or female. Krawczyk and Smyk (2016) have students judge the quality of academic papers, also telling the participants that the author is either male or female. Both studies find lower assessments of female instructors or researchers, respectively. A problem with the approach used in these two studies is that it is somewhat unnatural to explicitly withhold information on the exact speaker or researcher, and then provide a general level of gendered information (“a male author”). This may not just make the stimulus’ gender salient (which is good if we study gender effects), but may also provide respondents with cues regarding the goals of the study or the researcher, and what constitute a desirable answer (“experimenter demand,” Zizzo, 2010).

In two studies, we aim to probe these reports of gender bias. We hold any subjective-experience aspects across male and female stimuli constant by eliciting evaluations of teaching materials as suggested by Mengel et al.’s (2019) study. The controlled environment aims to isolate potential in-class effects from more fundamental aspects bias against female instructors. We control for the stimulus sampling problem (Wells and Windschitl, 1999) in MacNell et al. (2015) by using multiple stimuli for each gender, reducing the risk of uncontrolled variation within each gender. Other than in MacNell et al.’s (2015) study, there is also no past or current interaction between the evaluated instructors and participants. Moreover, we control for the potential demand effect of explicit gender information in Arbuckle and Williams (2003) and Krawczyk and Smyk (2016) by having gender enter in a subtle and fully natural way.

We test the Null hypotheses that no fundamental gender bias is observed if subjective experiences for raters with male and female instructors are eliminated, against the alternative hypotheses that biases persist in our design. Although the literature suggests a bias against female instructors, we use a more conservative two-sided hypothesis. Following the above-discussed literature, we test the hypothesis separately for male and female raters.

Hypothesis 1: Teaching materials are not evaluated based on the instructor’s gender.
Hypothesis 2: Male evaluators do not rate teaching materials based on the instructor’s gender.
Hypothesis 3: Female evaluators do not rate teaching materials based on the instructor’s gender.

In Studies 1 and 2, we test these hypotheses formally in a controlled experimental setting. Study 1 uses a laboratory setting, and Study 2 employs an online environment.

## Study 1

### Methods

The first study aims to test for gender bias in a typical laboratory student sample. Subjects were asked to browse through two short sets of teaching slides for an economics course on a computer screen. They were told that “These slides are used in a similar way for teaching purposes at the University of Heidelberg. The material of the slides is extracted from a lecture in the economics program of the University of Heidelberg (Bachelor).” This information is true. All subjects in an experimental session were presented with the same two sets of slides. The two sets were identical in terms of content, but differed in terms of layout. The content and the two different layouts that we used were held constant across all participating subjects and across all experimental sessions. On the title screen of the two sets of slides, the title of the lecture and the name of the lecturer were shown, as would naturally be the case for lecture notes. The surname of the lecturer was always “Müller,” a common German name. This surname was held constant across all subjects and across all sessions. The first name was identical on the two sets of slides and held constant for all subjects in a session, but was randomly varied across sessions, implementing the gender variation in a between-person design. In each session, the participants received the two sets of slides with either a female first name or a male first name. These names were selected from a list of the most popular male and female names for children born in Germany in the 1970s (see Supplementary Material, Part A). That is, these first names are not unusual nowadays for middle-aged professors and lecturers. Note that we do not analyze the effects of separate names. The stimulus sampling argument by Wells and Windschitl (1999) implies that there will be variation in how far any male or female name may be assessed positively or negatively. By using multiple names, we reduce this variation, looking at average effects over all names. By using fictitious names and by having subjects assess teaching material used at a different university, we ruled out that they associate the material with a real person, which might influence assessments.

After browsing the two slide sets, participants were asked to evaluate them along several dimensions. The first six items concerned the general quality of the slides: (1) clear structure; (2) clear content; (3) interesting topic; (4) mathematically sophisticated; (5) quality of the English language; and (6) suitability for independently studying the course. Three further questions concerned the comparison of the two layouts presented: (7) preference for a bright vs. dark background; (8) preference for a corporate vs. neutral design; (9) readability of the slides’ color scheme (links to slides and questionnaire are in Supplementary Material, Part A). Because our study aims to test differences in the effect of the between-person gender variation on quality assessment, we do not analyze the within-person layout variation addressed in questions 7–9. The variation and questions relating to layout only serve to provide the study background for the participants. Because these questions refer to the same first name on the slides, they cannot be used to study gender effects. The subjective judgments were not incentivized: There were no “correct” answers, thus incentivized judgments would have to be based on beliefs of others’ views (Krupka and Weber, 2013), which might be different from personal views. Moreover, while unincentivized questions may be noisier, it is not clear that complex incentive methods produce better data (Trautmann and Van de Kuilen, 2015). Incidentally, incentivization would be rather unnatural in the context of teaching evaluations.

In each session, all respondents were exposed to the same first name of an instructor. In addition, the two sets of slides that respondents were asked to browse and compare also featured the same name; they only differed in terms of layout. We applied a pen-and-paper style experiment, where subjects had to complete an evaluation sheet. On the sheet, before giving their evaluations, participants were first asked to indicate the name of the instructor given on the two sets of slides. They were told that this was needed to allow the researchers to unambiguously identify the slides the respondent saw, as there were different sets of slides (which is true given the variation in names across sessions). Participants also indicated their age, gender, and field of study.

While sample size was dictated by the subject pool availability, our sample is substantially larger than the other experimental studies (MacNell et al., 2015: 43 participants; Krawczyk and Smyk, 2016: 190 participants). In total 249 participants (118 men, 130 women, one subject did not indicate sex) with an average age of 23.45 (S.D. 5.32) participated at the University of Göttingen (Germany) experimental laboratory. On average, the experiment lasted 25 min. Subjects were paid a fee of €5 for participating in this experiment.

### Results

**FIGURE 1:**
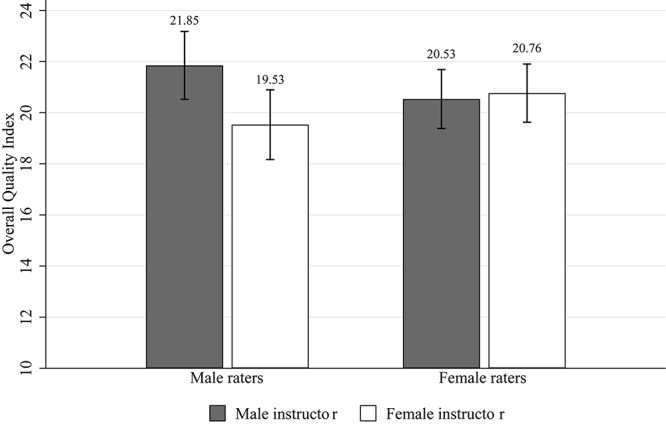
Overall quality index—Study 1. *Notes*: Quality index scores and standard error bars.

We form an index of the raters’ perceived quality of the lecture slides by summing up the six quality related items for each rater (the three layout items cannot be included as they refer to a comparison of the layouts of two sets by the same person; these items were only included to support the cover story). Subjects stated for each item an evaluation on a seven-points Likert scale which we scored 0 = “I do not agree” to 6 = “I agree.” The index corresponds to the sum of the six evaluations (Cronbach’s α = 0.62). Below we also give details for separate items. Table 1 and Figure 1 shows average index values for male and for female instructors, shown separately for all raters, male raters, and female raters, because Mengel et al. (2019) suggest that biased evaluations are driven by male raters. We drop three subjects in this analysis as they did not answer all questions encompassing our quality index. Although the literature speaks for a one-sided hypothesis, we report two-sided tests because it is conceivable that female raters hold more positive views of female instructors, and vice versa for males (see Study 2). In the raw comparisons, a *t*-test shows significantly higher evaluations of male instructors’ slides for male raters, and no significant difference for female raters. The difference for male raters is 2.32, which is 47% of the total standard deviation of the quality index of 4.90 in the full sample of all male and female raters and instructors. This effect is almost of medium effect size (Cohen’s *d* = 0.45) and it is larger than the one reported for teaching materials by Mengel et al. (2019).

**TABLE 1:** Overall quality index—Study 1.

	All raters	Male raters	Female raters
Male instructor, *N* = 113	21.15 (4.65)	21.85 (4.81)	20.53 (4.46)
Female instructor, *N* = 133	20.17 (5.08)	19.53 (5.46)	20.76 (4.70)
	*t*(244) = 1.56,	*t*(115) = 2.41,	*t*(126) = 0.29,
	*p* = 0.120	*p* = 0.018	*p* = 0.776

*Entries are values of the perceived quality index. Standard deviations are in parentheses.*

Table 2 shows the results of multivariate analyses. Columns (1) and (2) provide the estimation of regressions of the overall quality index on dummy variables for a female rater, a female instructor, and the interaction of female rater and female instructor. Model 1 includes no further controls. Model 2 includes controls for age, student status, and field of study. Table 2 reveals the substantially lower evaluations for female instructors. The positive interaction between female raters and female instructors indicates that the negative effect of female instructors is driven by male raters. Columns (3) and (4) provide the estimation of random effects panel regressions over all six items for each subject. That is, all six assessments by each rater enter separately, controlling for the intra-rater correlation. We again estimate the model with and without additional controls. Results replicate those for the overall index: both the negative effect for female raters and the positive interaction emerge significantly.

**TABLE 2:** Multivariate analysis—Study 1.

*Dependent*	(1) *Overall*	(2) *Overall*	(3) *Individual*	(4) *Individual*
*variable:*	*quality index*	*quality index*	*item responses*	*item responses*
Female rater	−1.32 (0.88)	−0.79 (0.90)	−0.23 (0.15)	−0.15 (0.15)
Female instructor	−2.32 (0.95)**	−2.33 (0.96)**	−0.38 (0.15)**	−0.38 (0.15)**
Female rater × female instructor	2.55 (1.25)**	2.40 (1.24)*	0.44 (0.21)**	0.42 (0.21)**
Controls	No	Yes	No	Yes
N raters/items (raters)	245	241	1485 (248)	1461 (244)

Columns (1) and (2) report OLS regression results, robust standard errors in parentheses. Columns (3) and (4) report panel regression results using six observations per rater. Controls are age, student status and field of study is economics, master program. * and ** indicate significance at the 10 and 5% level, respectively. Sample sizes vary across columns due to some missing values.

Table 3 provides the mean evaluation for male and female instructors on each of the six quality indicator items separately, with separate panels for male raters and female raters. There is a clear consistency for male raters: in all six items, the average score for male instructors’ slides is higher than for female instructors’ slides. For female raters, three items are rated higher and three items are rated lower for male instructors. Due to large variability in the evaluations, few within-item differences are statistically significant when analyzed separately. Significantly higher evaluations for male instructors by male raters are observed for clarity of structure and clarity of content. For females, female instructors’ slides are judged as more mathematically sophisticated. We conclude that male raters’ higher ratings for male instructors are highly consistent across items and not driven by a specific dimension of evaluation. On the other hand, female raters seem rather gender-unbiased in their evaluations. As a result, we can reject Hypotheses 1 and 2, but cannot reject Hypothesis 3 in Study 1.

**TABLE 3:** Separate item evaluations—Study 1.

					Quality of	Suitable for
	Clear	Clear	Interesting	Mathematically	English	independent
	structure	content	topic	sophisticated	language	study
***Male raters***						
Male instructor (*N* = 53)	3.89 (1.40)	4.02 (1.43)	3.36 (1.76)	3.68 (1.78)	4.26 (1.32)	2.64 (1.77)
Female instructor (*N* = 64)	3.16 (1.50)	3.53 (1.60)	2.98 (1.91)	3.53 (1.70)	4.09 (1.32)	2.23 (1.80)
	*t*(115) = 2.70,	*t*(115) = 1.72,	*t*(115) = 1.10,	*t*(115) = 0.46,	*t*(115) = 0.70,	*t*(115) = 1.23,
	*p* = 0.008	*p* = 0.089	*p* = 0.276	*p* = 0.648	*p* = 0.488	*p* = 0.222
***Female raters***						
Male instructor (*N* = 60)	3.95 (1.36)	3.67 (1.34)	2.48 (1.72)	3.75 (1.72)	4.12 (1.40)	2.57 (1.75)
Female instructor (*N* = 68)	3.78 (1.24)	3.88 (1.54)	2.53 (1.80)	4.29 (1.61)	3.78 (1.56)	2.5 (1.67)
	*t*(126) = 0.74,	*t*(126) = −0.84,	*t*(126) = −0.15,	*t*(126) = −1.84,	*t*(126) = 1.28,	*t*(126) = 0.22,
	*p* = 0.460	*p* = 0.402	*p* = 0.883	*p* = 0.067	*p* = 0.204	*p* = 0.826

*Entries are mean evaluations and standard deviations in parentheses.*

## Study 2

In study 1, we replicated previous findings of more positive evaluations of teaching material, excluding any potential spillover effects from classroom experience. We thus support the idea of direct gender bias, not related to any direct teaching experience with the person assessed by the rater, as previously suggested by MacNell et al. (2015). As in Mengel et al. (2019), we find effects driven by male raters. However, we do find some suggestive evidence that females may sometimes be evaluated better by female raters. In Study 2, we aim to generalize these analyses from our German student sample to a larger and more diverse subject pool in an online experiment on Amazon Mechanical Turk (MTurk).

### Methods

Following the same paradigm as in Study 1, respondents are asked to evaluate a paired set of teaching slides with identical content and two different layouts, with a between-person variation in the gender signaled by the first name of the instructor mentioned on the slides. To study the generality of the findings in Study 1, Study 2 makes use of a large sample of participants recruited through Amazon MTurk. Respondents browsed the slides and provided their assessments online using oTree (Chen et al., 2016; screenshot in Supplementary Material, Part B). The pool of respondents was restricted to participants in the United States, and thus a set of common male and female American names in the 1970s were used for the instructors (see Supplementary Material, Part A). Because the original set of slides was considered too technical for a mainly non-economics and partly non-academic pool at MTurk, and too long for the online format, we used a shorter and less technical set of slides that describes the different functions of financial intermediaries like banks. As in Study 1, respondents were told that these slides were used in a similar form in the bachelor economics program at the University of Heidelberg (Germany), which is true. There was still some mathematical content in the slides (see Supplementary Material, Part A), allowing us to keep the set of evaluation questions identical to Study 1.

To make the gender manipulation salient, at the beginning of the evaluation part, respondents were asked to type in the name of the instructor mentioned on the slides to identify the set they saw, because there were different sets. The assessment questionnaire and the resulting variables were identical to the one used in Study 1. Power calculations based on our results in Study 1 show that, to obtain an 80% power to detect the effect found in Study 1 in the full population, a sample size of 79 per cell (instructor gender X rater sex) is sufficient. We run a substantially larger total sample size of 804 participants (414 men, 386 women, one other gender, three did not reveal their sex; cells between 178 and 209 participants), to allow exploratory analysis of the subgroups of interest (those with college degree and those without) with sufficient power as calculated. The average age of the participants was 36.6 (SD 11.29), 345 held a Bachelor degree, and 125 were current students. Participants received a fixed remuneration of $0.75 for their participation in the study.

### Results

**FIGURE 2:**
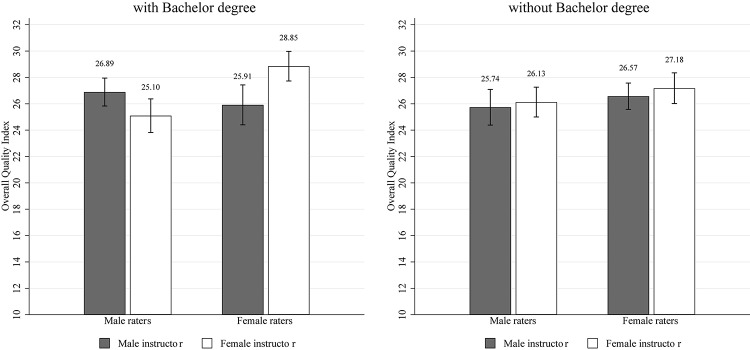
Overall quality index (conditional on Bachelor degree)—Study 2. *Notes*: Quality index scores and standard error bars. The left (right) panel presents the data of participants with (without) a Bachelor’s degree.

Table 4 and Figure 2 show results for the overall quality index as in Table 1 and Figure 1 (Cronbach’s α = 0.82). The table shows results for the full sample, as well as exploratory results for participants with a completed Bachelor degree and participants without a completed Bachelor degree. The former subsample is closer to Study 1 in that participants (1) were college students before and may thus share typical experiences with our pool in Study 1, and (2) may more easily relate to teaching materials and to assessing their content in the style of a typical teaching evaluation. Table 4 provides two insights. First, in those without a college degree, no gender biases are observed. Second, in those with a college degree, we replicate the effect of male raters assessing male-named slides more favorable. It turns out that we find a small effect size (Cohen’s *d* = 0.31). However, in contrast to Study 1 where females were more balanced overall, we now observe that female raters assess female-named slides more favorable. Hence, in the online study, we do confirm the gender bias in male college educated raters, but also find a bias in female college educated raters, giving higher ratings to women. Interestingly, the latter effect is of stronger magnitude (Cohen’s *d* = 0.49) and of medium size.

**TABLE 4:** Overall quality index—Study 2.

	All raters	Male raters	Female raters
***Full sample***		*N = 414*	*N = 386*
Male instructor, *N* = 419	26.32 (6.18)	26.33 (6.23)	26.31 (6.17)
Female instructor, *N* = 385	26.65 (6.07)	25.65 (6.12)	27.74 (5.83)
	*t*(802) = −0.77,	*t*(412) = 1.11,	*t*(384) = −2.33,
	*p* = 0.443	*p* = 0.269	*p* = 0.020
***Bachelor degree: yes***		*N = 201*	*N = 142*
Male instructor, *N* = 190	26.46 (6.13)	26.89 (5.52)	25.91 (6.88)
Female instructor, *N* = 155	26.61 (5.87)	25.10 (6.24)	28.85 (4.34)
	*t*(343) = −0.228, *p* = 0.820	*t*(299) = 2.16, *p* = 0.032	*t*(140) = −2.91, *p* = 0.004
***Bachelor degree: no***		*N = 213*	*N = 244*
Male instructor, N = 229	26.21 (6.23)	25.74 (6.88)	26.57 (5.67)
Female instructor, *N* = 230	26.68 (6.21)	26.13 (6.01)	27.18 (6.39)
	*t*(457) = −0.82,	*t*(211) = −0.44,	*t*(242) = −0.79,
	*p* = 0.411	*p* = 0.646	*p* = 0.433

*Entries are values of the perceived quality index. Standard deviations are in parentheses.*

To obtain a further understanding of the relationship of these results with those of the student sample in Study 1, we run exploratory analyses also for the sample of current students in the MTurk sample. Clearly, this sample has low power according to our power analysis and the results are at best suggestive. Results show that in the current student sample the gender bias for male raters emerges as in Study 1, with no clear effect for female raters (Supplementary Material, Part C).

Table 5 probes the robustness of the exploratory results in a multivariate analysis with and without controlling for age and background in economics. The raw results emerge consistently also in the multivariate analysis of the overall quality index and panel regressions considering each item separately. Again, no effects are observed for those without a college degree. The differential effect of college also replicates in a full multivariate analysis of the full sample with college as an interaction term (Supplementary Material, Part C). We also ran a robustness check where we control for whether MTurk subjects actually browsed the slides. Results replicate the effects for the academic sample and the absence of any effects in the non-academic sample (Supplementary Material, Part C). As a result, we can reject Hypotheses 2 and 3 for the academic sample in Study 2. For the non-academic sample, we cannot reject any of the Hypotheses 1–3.

**TABLE 5:** Multivariate analysis—Study 2.

*Dependent*	(1) *Overall*	(2) *Overall*	(3) *Individual*	(4) *Individual*
*variable:*	*quality index*	*quality index*	*item responses*	*item responses*
***Bachelor degree: yes***				
Female rater	−0.97 (0.93)	−0.85 (0.93)	−0.16 (0.14)	−0.14 (0.15)
Female instructor	−1.79 (0.84)**	−1.79 (0.84)**	−0.30 (0.14)**	−0.30 (0.14)**
Female rater × female instructor	4. 73 (1.26)***	4.69 (1.26)***	0.79 (0.22)***	0.78 (0.22)***
Controls	No	Yes	No	Yes
N	343	343	2058 (343)	2058 (343)
***Bachelor degree: no***				
Female rater	0.84 (0.85)	0.66 (0.94)	0.14 (0.14)	0.11 (0.15)
Female instructor	0.39 (0.89)	0.66 (0.97)	0.07 (0.14)	0.11 (0.15)
Female rater × female instructor	0.22 (1.18)	−0.02 (1.29)	0.04 (0.19)	0.00 (0.21)
Controls	No	Yes	No	Yes
N raters / items (raters)	457	394	2742 (457)	2364 (394)

Columns (1) and (2) report OLS regression results, robust standard errors in parentheses. Columns (3) and (4) report panel regression results using six observations per rater. Controls are age and indicator if respondent has some background/experience in economics. ** and *** indicate significance at the 5 and 1% level, respectively. Sample sizes vary across columns due to some missing values.

## Discussion

Study 1 finds differences in the evaluation of identical teaching slides depending on the gender signaled by the instructor’s name. That is, any confounding effects of the class experience that are otherwise unrelated to the teaching effectiveness, and potentially affect teaching evaluations, are excluded by design in our study. Consistent with previous studies, female instructors receive worse evaluations than male instructors when assessed by male raters. One explanation is that the evaluation process is affected by gender stereotypes: male raters may believe that female instructors perform worse in technical fields like economics (Heinz et al., 2016; Boring, 2017), and assessments are affected by such beliefs even in the absence of differences. Females hold somewhat more positive views of female instructors. These results support an interpretation of previous findings in terms of a true gender bias in teaching evaluations.

Study 2 showed that the gender bias in the assessment of mere teaching material replicates in an online experiment in a sample of older MTurk participants with a college degree. In contrast to Study 1, however, both males and females assess instructors of their own gender more favorable. Restricting the analyses to an arguably underpowered sample of current students among the MTurk participants yields results identical to Study 1, with a bias observed for male raters but not for female raters. On the other hand, those without a college degree show no gender bias at all (with substantial power to identify an effect of the size observed in Study 1). These findings of the exploratory subgroup analysis suggest two conclusions. First, there is no wholesale gender bias of everybody simply rating work of females worse than that of males. In particular, past experiences made in university classes seems important for the bias in teaching materials to emerge. We refer to these effects as “study experience.” The study-experience effect we find may for example derive from past in-class experiences at college where staff is predominantly male. In contrast, those without college may associate educational material with educational contexts that are not dominated by males (high school, adult education).

Second, biased evaluations against female instructors in our studies only obtain for male raters. In Study 1, we find suggestive evidence that female raters give better evaluations to female instructors (mathematical sophistication), and in Study 2, more favorable evaluations by female raters for female instructors strongly emerge for those with college education. The evidence is consistent with recent results by Funk et al. (2019) who argue that female subjects may favor female instructors when the pool of instructors is male-dominated. Moreover, Rudman and Goodwin (2004) show that in-group bias may be strong in women; it may thus overcome other biases if they existed. However, it is also important to note that the male rater bias toward better evaluations for male instructors is, across all analyses in the paper, the one that consistently emerges. This is consistent with the widely observed gender effect in teaching evaluations that we aimed to shed light on.

Previous research has demonstrated that lower teaching evaluations for women do not seem to be related to aspects of teaching measurable by students’ study activity or success. However, it is conceivable that certain aspects of teaching by women are in fact perceived as less pleasant, in particular by male students. For example, Arbuckle and Williams (2003) showed that aspects of voice tone may indeed be judged with a gender bias. Building on work by MacNell et al. (2015), the current study excluded any such aspects by having people judge pure teaching material. Despite this fact and despite a very subtle gender identification on the materials, we find clear evidence that identical teaching material may be judged differently if it comes from male or female instructors. As in Mengel et al. (2019), we find that more negative assessments for females are driven by male raters in a laboratory experiment with a student sample. For an online sample of raters with a college degree we find biases toward their own gender for both male and female raters. For a broader set of non-college respondents in the online sample, no biases are observed. That is, despite the absence of any class-room experience confounds, the bias seems to be related to current study experience. We can still consider it a bias though, as any extrapolation from other courses or experiences would be unwarranted in the current settings with the evaluation of identical teaching materials.

Overall, our results suggest that gender biases may be important in teaching evaluations if they even emerge in the most reduced contexts without any personal interaction and possible softer factors entering the assessment. The results question the validity of teaching evaluations as a governance tool used in hiring and promotion decisions. The findings in Arbuckle and Williams (2003); MacNell et al. (2015), and the current paper that even supposedly objective materials may not be judged in a neutral way if gender information is available may be relevant beyond the narrow domain studied here. For example, a recent study by Brock and De Haas (2020) investigates whether loan applications are judged differently when coming from male or female business holders. Real loan applications involve personal interaction that allows loan officers to obtain soft information about the applicant, but may also lead to biases if personality aspects related to gender may inappropriately influence decisions. Eliminating the confounding channel of soft borrower information similar to the current setting, Brock and De Haas (2020) show that biases can emerge already in the assessment of gender-named loan application files. They show that experience effects and implicit biases may be of relevance for such assessment biases. Our study suggests that future research on biases in teaching evaluations may also benefit from exploring these channels.

## Data Availability Statement

All data, analyses, and other materials necessary for replication of the current studies are available online at the OSF repository https://osf.io/x298e/.

## Ethics Statement

### Study 1 (Laboratory)

In accordance with the Declaration of Helsinki, all participants were requested to read an online consent form and agreed with its terms (by clicking) before registering to take part in an experiment. Participants were guaranteed the anonymity of the data generated during the experiment. Subjects could only take part in the study, after they gave consent, by clicking the OK button. Thus, all subjects who participated in our Study 1 gave the written informed consent. At the University of Göttingen, there is no internal review board. All experimental protocols were approved through the experimental laboratory at the University of Göttingen (GLOBE Lab).

### Study 2 (MTurk)

In accordance with the Declaration of Helsinki, all participants were requested to read an online consent form and agreed with its terms (by clicking) before registering to take part in an experiment. At the University of Göttingen, there is no internal review board. All experimental protocols were approved through the experimental laboratory at the University of Göttingen (GLOBE Lab).

## Author Contributions

The authors contributed equally to the research. AÖ, HR, and ST designed Study 1 and wrote the manuscript. AÖ, HR, ST, and CK-K designed Study 2. AÖ and HR conducted the experiment in Study 1. CK-K conducted the experiment in Study 2. AÖ and CK-K prepared the data. AÖ and ST analyzed the data.

## Conflict of Interest

The authors declare that the research was conducted in the absence of any commercial or financial relationships that could be construed as a potential conflict of interest.
