# Supplementary material for: Gender Bias in the Evaluation of Teaching Materials
Source: Front Psychol. 2020 May 26;11:1074. doi: 10.3389/fpsyg.2020.01074 (PMC7326140; doi:10.3389/fpsyg.2020.01074)
Supplement: Supplementary file 1 [file Data_Sheet_1.pdf]

Supplementary Material

1 Part A

1.1 Study 1

The full questionnaire (in English and German), the lecture slides we used, and all other replication materials can be found at the OSF repository https://osf.io/x298e/?view_only=f9801d89a1424879b8360345b54756f7.

Names were retrieved from https://www.beliebte-vornamen.de/3774-1970er-jahre.htm: Claudia, Katrin, Nicole, Sandra, Stefanie, Tanja, Anja; Andreas, Christian, Marcus, Matthias, Michael, Sven, Jan.

1.2 Study 2

The study was preregistered on aspredicted.org as #24898: http://aspredicted.org/blind.php?x=ie3bk5. All replication materials can be found at the OSF repository https://osf.io/x298e/?view_only=f9801d89a1424879b8360345b54756f7.

Names were retrieved from https://www.ssa.gov/oact/babynames/decades/names1970s.html: Christina, Jennifer, Jessica, Julie, Lisa, Michelle, Nicole, Sarah, Stephanie, Susan; Brian, Christopher, David, John, Mark, Matthew, Michael, Richard, Robert, Thomas.

We provide the following supporting information: Participants on average took 3:50 minutes to complete the experiment and received $0.75. The corresponding average hourly pay would be $7.71. We restricted participation to US Citizens only, but did not require any other qualifications. 1222 Participants accepted the assignment, 804 (65.8%) completed it. Of the 418 participants who did not complete the experiment, 141 (33.7%) dropped out on the instructions page, 258 (61.7%) on the main task, and the remaining 19 (4.5%) on a later page. Of the 277 who dropped out on the main task page or later 142 (51.3%) saw a female instructor name and 135 (48.7%) a male instructor name, indicating that attrition is not treatment specific.

2 Part B

In Study 2, the two sets of slides were shown side by side on participants' screens. They could navigate the sets independently using the buttons below. Questions regarding the slides were shown on the same screen (it was required to scroll down the page to reveal all questions). Figure B1 provides a screenshot.

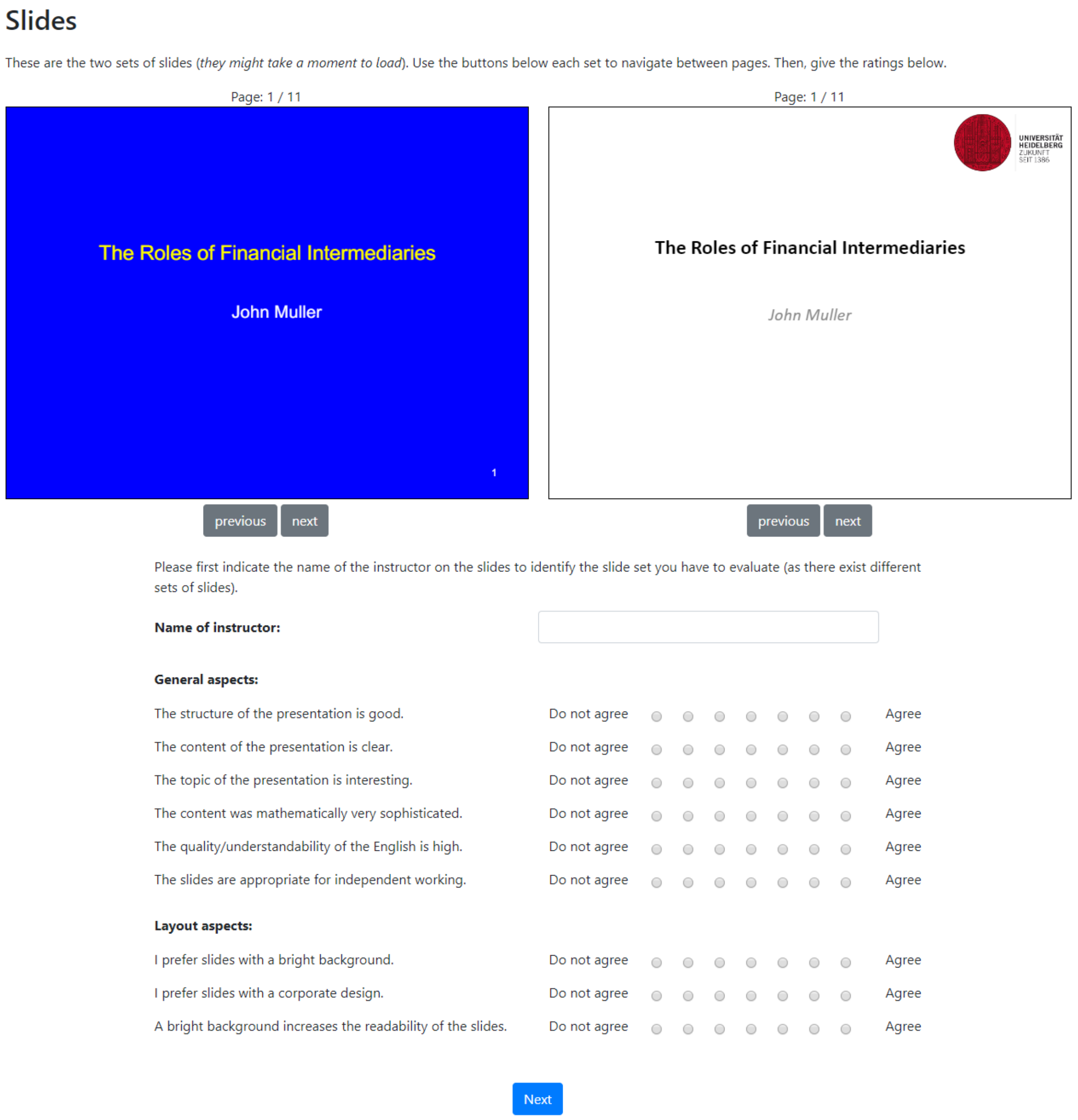

Supplementary Figure 1. Study 2 screenshot

3 Part C

Table C1. Multivariate Analysis – Study 2
(Full Sample with Interaction Terms for Bachelor Degree)

Dependent variable:	(1) *Overall Quality Index*	(2) *Overall Quality Index*	(3) *Individual item responses*	(4) *Individual item responses*
Female rater	0.84 (0.85)	0.73 (0.93)	0.14 (0.14)	0.12 (0.16)
Female instructor	0.39 (0.89)	0.69 (0.97)	0.07 (0.15)	0.11 (0.16)
Female rater × female instructor	0.22 (1.18)	-0.09 (1.29)	0.04 (0.20)	-0.02 (0.21)
Bachelor	1.15 (0.87)	1.36 (0.91)	0.19 (0.14)	0.23 (0.15)
Bachelor x fem. rater	-1.81 (1.26)	-1.74 (1.31)	-0.30 (0.21)	-0.29 (0.22)
Bachelor x fem. instr.	-2.18 (1.22)*	-2.48 (1.28)*	-0.36 (0.20)*	-0.41 (0.21)*
Bachelor. x fem. rater x fem instr.	4.51 (1.73)***	4.83 (1.80)***	0.75 (0.29)***	0.81 (0.30)***
Controls	No	Yes	No	Yes
N raters / items (raters)	800	737	4800 (800)	4422 (7437)

Notes: Columns (1) and (2) report OLS regression results, robust standard errors in parentheses. Columns (3) and (4) report panel regression results using 6 observations per rater. We include "Bachelor", a dummy variable, which is positive for bachelor students. Moreover, we interact our independent variables with "Bachelor." Controls are age and indicator if respondent has some background / experience in economics. *, *** indicates significance at the 10%, 1% level, respectively.

Table C2. Multivariate Analysis – Study 2 – Robustness Check

Dependent variable:	(1) *Overall Quality Index*	(2) *Overall Quality Index*	(3) *Individual item responses*	(4) *Individual item responses*
Bachelor degree: yes				
Female rater	-0.83 (0.95)	-0.73 (0.95)	-0.14 (0.15)	-0.12 (0.15)
Female instructor	-1.76 (0.85)**	-1.77 (0.85) **	-0.29 (0.14)**	-0.30 (0.14)**
Female rater × female instructor	4.67 (1.26)***	4.64 (1.26)***	0.78 (0.22)***	0.77 (0.22)***
Controls for both sets of slides browsed	Yes	Yes	Yes	Yes
Controls	No	Yes	No	Yes
N	343	343	2058 (343)	2058 (343)
Bachelor degree: no				
Female rater	0.83 (0.85)	0.66 (0.93)	0.14 (0.14)	0.11 (0.15)
Female instructor	0.36 (0.88)	0.63 (0.97)	0.06 (0.14)	0.10 (0.16)
Female rater × female instructor	0.20 (1.18)	-0.04 (1.29)	0.03 (0.20)	-0.01 (0.21)
Controls for both sets of slides browsed	Yes	Yes	Yes	Yes
Controls	No	Yes	No	Yes
N	457	394	2742 (457)	2364 (394)

Notes: Columns (1) and (2) report OLS regression results, robust standard errors in parentheses. Columns (3) and (4) report panel regression results using 6 observations per rater. Controls are age and indicator if respondent has some background / experience in economics. **, *** indicates significance at the 5%, 1% level, respectively. Sample sizes vary across columns due to some missing values.

Table C3. Overall Quality Index – Study 2 (Current Students)

	All raters	Male raters (N=72)	Female raters (N=52)
Male instructor, N=64	27.53 (6.60)	28.26 (6.04)	26.40 (7.38)
Female instructor, N=61	26.28 (5.90)	25.21 (6.72)	27.30 (4.47)
	t(123)=1.12, p=0.266	t(70)=2.02, p=0.047	t(50)=-0.53, p=0.596

Notes: Entries are values of the perceived quality index. Standard deviations in parentheses.

Table C4. Multivariate Analysis – Study 2 (Current Students)

Dependent variable:	(1) *Overall Quality Index*	(2) *Overall Quality Index*	(3) *Individual item responses*	(4) *Individual item responses*
Female rater	-1.86 (1.76)	-1.98 (1.78)	-0.31 (0.27)	-0.33 (0.27)
Female instructor	-3.04 (1.52)**	-3.25 (1.56)**	-0.51 (0.25)**	-0.54 (0.25)**
Female rater × female instructor	3.94 (2.28)*	4.26 (2.32)*	0.66 (0.38)*	0.71 (0.38)*
Controls	No	Yes	No	Yes
N	124	124	744 (124)	744 (124)

Notes: Columns (1) and (2) report OLS regression results, robust standard errors in parentheses. Columns (3) and (4) report panel regression results using 6 observations per rater. Controls are age and indicator if respondent has some background / experience in economics. *, ** indicates significance at the 10%, 5% level, respectively.
